# Prediction of Potential Distribution of *Carposina coreana* in China under the Current and Future Climate Change

**DOI:** 10.3390/insects15060411

**Published:** 2024-06-03

**Authors:** Guolei Zhang, Sai Liu, Changqing Xu, Hongshuang Wei, Kun Guo, Rong Xu, Haili Qiao, Pengfei Lu

**Affiliations:** 1 State Key Laboratory of Efficient Production of Forest Resources, College of Forestry, Beijing Forestry University, Beijing 100083, China; zguolei2021@163.com; 2 Institute of Medical Plant Development, Chinese Academy of Medical Sciences and Peking Union Medical College, Beijing 100193, China; liusaimail@163.com (S.L.); cqxu@implad.ac.cn (C.X.); hswei@implad.ac.cn (H.W.); kguo@implad.ac.cn (K.G.); rxu@implad.ac.cn (R.X.)

**Keywords:** *Carposina coreana*, climate change, suitable area, MaxEnt

## Abstract

**Simple Summary:**

*Carposina coreana* Kim is the most serious pest of *Cornus officinalis*. In recent years, its damage to *C. officinalis* has become increasingly serious, causing enormous economic losses in China. Here, the maximum entropy (MaxEnt) model was used to predict the distribution of *C. coreana* under current climate scenarios and future climate scenarios in China with ArcGIS software. Suitable areas for *C. coreana* under the current climate scenarios were mainly distributed in central China, and the highly suitable areas were distributed in southern Shaanxi, southwestern Henan, and northwestern Hubei. Under future climate scenarios, the boundaries of the suitable areas for *C. coreana* tended to shift to northern China. Given the predictive results of this study, we can clearly see the future diffusion trend of *C. coreana* in China, which has important theoretical significance for the control of this pest in China.

**Abstract:**

*Carposina coreana* is an important pest of *Cornus officinalis*, distributed in China, Korea, and Japan. In recent years, its damage to *C. officinalis* has become increasingly serious, causing enormous economic losses in China. This study and prediction of current and future suitable habitats for *C. coreana* in China can provide an important reference for the monitoring, early warning, prevention, and control of the pest. In this study, the potential distributions of *C. coreana* in China under current climate and future climate models were predicted using the maximum entropy (MaxEnt) model with ArcGIS software. The distribution point data of *C. coreana* were screened using the buffer screening method. Nineteen environmental variables were screened using the knife-cut method and variable correlation analysis. The parameters of the MaxEnt model were optimized using the kuenm package in R software. The MaxEnt model, combined with key environmental variables, was used to predict the distribution range of the suitable area for *C. coreana* under the current (1971–2000) and four future scenarios. The buffer screening method screened data from 41 distribution points that could be used for modeling. The main factors affecting the distribution of *C. coreana* were precipitation in the driest month (Bio14), precipitation in the warmest quarter (Bio18), precipitation in the coldest quarter (Bio19), the standard deviation of seasonal variation of temperature (Bio4), minimum temperature in the coldest month (Bio6), and average temperature in the coldest quarter (Bio11). The feature class (FC) after the kuenm package optimization was a Q-quadratic T-threshold combination, and the regularization multiplier (RM) was 0.8. The suitable areas for *C. coreana* under the current climate model were mainly distributed in central China, and the highly suitable areas were distributed in southern Shaanxi, southwestern Henan, and northwestern Hubei. The lowest temperature in the coldest month (Bio6), the average temperature in the coldest quarter (Bio11), and the precipitation in the warmest quarter (Bio18) all had good predictive ability. In future climate scenarios, the boundary of the suitable area for *C. coreana* in China is expected to shift northward, and thus, most of the future climate scenarios would shift northward.

## 1. Introduction

**Figure 1:**
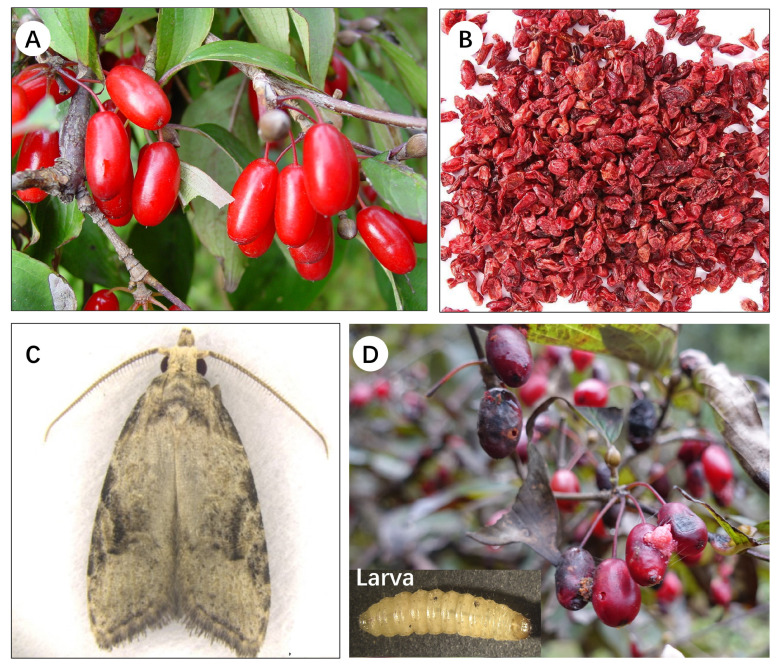
*Cornus officinalis* and the symptoms of fruits harmed by *Carposina coreana*. (**A**) Mature fruits of *C. officinalis*. (**B**) Dried ripe fruit peels of *C. officinalis*. (**C**) Adult of *C*. *coreana*. (**D**) Harmful symptoms of *C*. *coreana* larva on fruits of *C. officinalis*.

**Figure 2:**
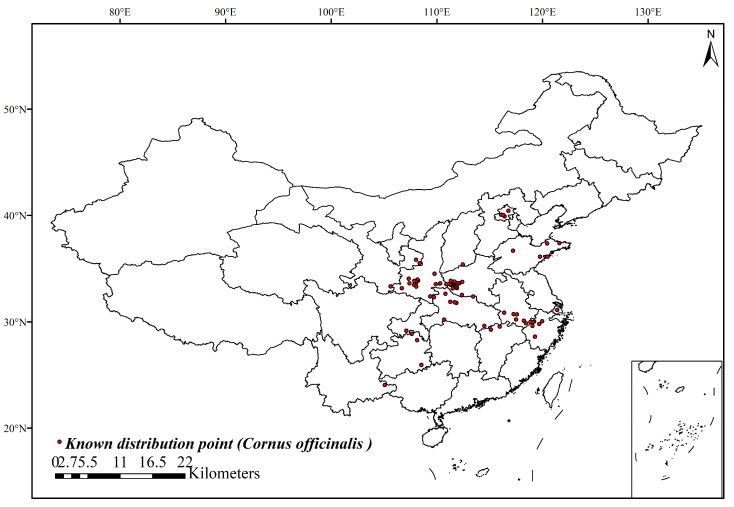
Known geographical distribution records of *Cornus officinalis* in China.

*Cornus officinalis* Sieb. et Zucc.,1835 (Shanzhuyu) (Cornaceae) is a deciduous tree originating from eastern Asia, mainly distributed in China, Japan, and South Korea, which is commonly used in traditional medicine (Figure 1A) [1]. The dried ripe fruit peels of *C. officinalis* (Figure 1B) are rich in iridoid glycosides, saponins, polysaccharides, ursolic acid, oleanolic acid, and tannins [2]. Studies have proven that consuming the berry peels can regulate the human immune system and provide anti-aging, anti-inflammatory, and anti-shock effects [2]. In recent years, the demand for the dried ripe fruit peels of *C. officinalis* has been growing domestically and internationally, which has led to increasing requirements for high yield and quality. The planting area of this herb is expanding year by year and is mainly distributed across 11 provinces, including Zhejiang, Henan, Shaanxi, Shanxi, and Anhui (Figure 2) [3].

*Carposina coreana* Kim, 1955 (Lepidoptera: Carposinidae) is the most serious fruit-boring insect pest of *C. officinalis*. The adult *C. coreana* insect is grey, similar in color to the trunk of *C. officinalis*, with a body length of 6.8–9.5 mm. The linear antenna is about half the length of its forewings (Figure 1C) [4]. The body of the larva is light yellow, with obvious hairy patches on each body segment, and no hip segments. There is an obvious “Y” shaped pattern on the front of the head (Figure 1D) [4]. The larvae penetrate the fruit of *C. officinalis* and eat the flesh. The empty fruit is covered by the feces of the larvae, leaving only a layer of epidermis, causing the fruit to lose its medicinal value (Figure 1D). Like other monophagous pests, *C. coreana* has a specific host plant, and solely feeds on the plant of *C. officinalis*. This pest is mainly distributed in Shaanxi, Henan, Zhejiang, and Shanxi in China, and it is also found in Korea and Japan [4,5]. Once the fruit of *C. officinalis* is damaged by this pest, the insect infestation rate can reach 70–80%. If the pest causes damage without control, the insect infestation rate can even reach 90% or higher. The pest can cause a decrease of about 30% in fruit yield, or even a complete loss of harvest [6]. In recent years, the rate of fruit damage caused by *C. coreana* has been increasing year by year in newly introduced and cultivated areas, such as Anhui and Hubei. The potential risk of damage from these pests is very high. If not controlled, once the pest population spreads, it poses a huge threat to the cultivation and industrial development of *C. officinalis*. Therefore, to reduce the risk of further spread of *C. coreana* into other regions, it is crucial to predict its potential distribution areas and identify the environmental factors that affect its distribution.

Studies have shown that the distribution of species is affected by climate change, which also affects the richness and composition of species by altering temperature and humidity [7]. Temperature and humidity are key factors that determine habitat range and population size [8,9], as they not only affect the growth and development of insects, but also interfere with the growth and development of insect host plants, indirectly affecting the spread of insect populations [10,11]. On the one hand, temperature is the most important factor in determining insect growth and development rate. As ectothermic animals, insects completely rely on external temperature to complete their growth and development, as well as various physiological activities. Therefore, climate warming is likely to have direct or indirect effects on insect individuals, populations, communities, and food webs in various ways [12]. Within a suitable range, an increase in temperature can accelerate the growth and development rate of insects, but when the temperature exceeds a certain threshold, the speed of insect development will rapidly decline or even stop [13]. On the other hand, changes in environmental humidity often directly affect the balance of water in an insect’s body. Excessive or insufficient humidity can cause discomfort or affect feeding and other normal physiological functions, thereby affecting the occurrence of different stages of individual development and even entire populations [14,15]. Therefore, when predicting suitable habitats for *C. coreana*, environmental factors such as temperature and humidity are mainly considered.

The ecological niche model utilizes the known distribution data of species and related climate variables to calculate the actual and potential distribution of species in other times and spaces through certain algorithms [16]. Common models used for forecasting suitable habitats for invasive alien species include the maximum entropy (MaxEnt) [17,18], genetic algorithm for rule-set production (GARP) [19], CLIMEX [20,21], and boosted regression tree (BRT) [22] models. The MaxEnt model is the optimal prediction method for predicting potential habitats based on geographic information data of existing species occurrence locations, and has better predictive accuracy than other models [23,24]. Therefore, the MaxEnt model has been widely used in prediction research for various types of biological habitats, such as those for plants, insects, fungi, etc. In recent years, there have been reports on the spread of suitable habitats for *Wasmannia auropunctata* Roger, 1863 (Hymenoptera: Formicidae) [25], *Stipa purpurea* Griseb., 1868 (Poaceae) [26], *Taxus wallichiana* Florin, 1948 (Taxaceae) [27], *Pennisetum alopecuroides* Spreng, 1825 (Poaceae) [28], and *Ceratothripoides claratris* (Shumsher), 1946 (Thysanoptera: Thripidae) [29].

At present, the research on *C. coreana* mainly focuses on the occurrence of hazards, morphological characteristics [4,5], and biological characteristics [30]. There have been no studies on the Chinese scale to predict the distribution of *C. coreana* habitats. Based on the data of insect distribution points and important environmental variables affecting insect distribution, this study used the MaxEnt model to predict suitable habitats for *C. coreana* in China under current and future climate conditions. Our results also provide theoretical references for formulating policies for *C. coreana* management and control.

## 2. Materials and Methods

### 2.1. Collection and Processing of Distribution Data

Data comprising a total of 41 distribution points for *C. coreana* were used in this study, obtained from two sources: (1) from the literature (six distribution points were obtained); and (2) from field investigation and collection. During the period from 2015 to 2023, field investigations were conducted on the distribution areas of *C. coreana* in China. The samples collected in this study included adults and larvae. They were identified through morphological and molecular markers, and a total of 35 distribution points were obtained in this manner. The research materials in this study have been stored at the Institute of Medical Plant Development, Chinese Academy of Medical Science.

To prevent the overfitting of the distribution points data, the distribution points were filtered by ArcGIS buffer analysis with a buffer radius of 5 km, ensuring that only one distribution point was retained within this buffer radius range [31].

### 2.2. Obtaining and Filtering Environment Variables

Bioclimatic variables have been widely used to predict the distribution of invasive alien insects at regional and global scales [23]. The environmental variables used in this study were all from the Worldclim database (www.worldclim.org accessed on 24 February 2023) with a spatial resolution of 2.5 arcmin. The version of the data used was version 2.1 [32]. Historical climate data were selected for the environmental variables from 1971 to 2000. The future climate data were selected for four time periods: 2021–2040, 2041–2060, 2061–2080, and 2081–2100 [31].

Future climate data were selected from the Beijing Climate Center Climate System Model 2 Medium Resolution (BCC-CSM2-MR) in the 6th International Coupled Model Intercomparison Project Phase 6 (CMIP6) [33]. The future climate data of CMIP6 had four shared socioeconomic pathways: SSP126, SSP245, SSP370, and SSP585. The scenario of shared socioeconomic pathways was set to obtain specific socio-economic development scenarios based on the current national and regional realities, as well as development plans. SSP126 belonged to the low-forcing category. SSP245 belonged to the medium-forcing category. SSP370 and SSP585 belonged to the high-forcing category. The four scenarios assumed that the radiative forcing of the year 2100 stabled at 2.6, 4.5, 7.0, and 8.5 W/m^−2^ [34].

Excessive environmental variables hurt model construction, so it was necessary to filter environmental variables to reduce redundant information and obtain the most favorable environmental variables for the distribution of *C. coreana*. Pearson correlation analysis was conducted on 19 environmental variables using SPSS software. If the absolute value of correlation between the two environmental variables was |r| > 0.8, only one environmental variable was selected for the model. Finally, the key environmental variables were screened based on their ecological significance as environmental variables for the studied species.

### 2.3. Software and Map Data

The MaxEnt software (version 3.4.1) used in this study was downloaded from the MaxEnt homepage [35]. ArcGIS 10.8, developed by the Environmental Systems Research Institute of the Unites States, was used [31]. The 1:4 million scale vector map of China was downloaded from the Resource and Environment Science and Data Center of the Chinese Academy of Sciences.

### 2.4. Predicting Suitable Areas for C. coreana Using MaxEnt Model

#### 2.4.1. Setting and Optimization of MaxEnt Model Parameters

The processed *C. coreana* sample points and climate variables were imported into the MaxEnt model, which was set with the following parameters: The output format of model was selected as “Logistic” [36], the output file type was selected as “Asc” [37], and “Subsample” was selected for repeated iteration. The corresponding curve and the jack-knife method results were used to determine the key environmental factors affecting *C. coreana*.

The MaxEnt software provided a set of parameters by default when constructing a distribution model, but this set of default parameters was affected by the test data. The model under the default parameters was sensitive to the test data, and was very prone to overfitting. The prediction results of the model often led to significant differences from the actual distribution [38]. Therefore, model parameter optimization was crucial for the accuracy of predicting the suitable growth area of *C. coreana*.

**Figure 3:**
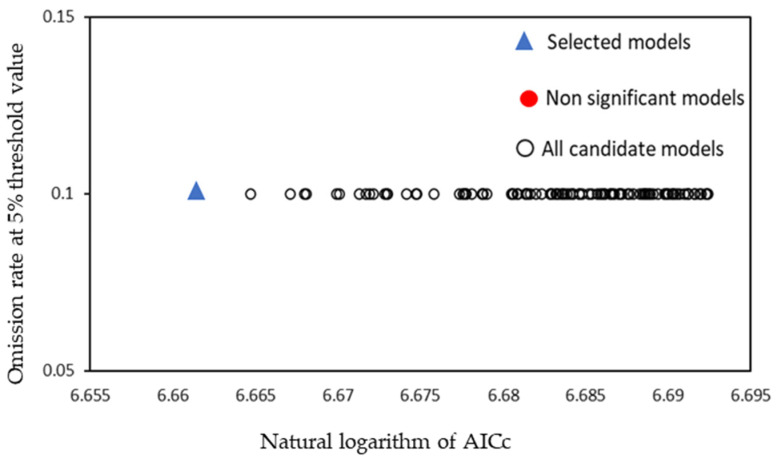
Results of kuenm package in R, omission rates, and AICc values for all, non-significant, and selected “best” candidate models for *Carposina coreana*. Models were selected based on statistical significance, omission rates, and AICc criteria.

In this study, two important parameters in MaxEnt were optimized by calling the kuenm package in the R software: feature class (FC) and regularization multiplier (RM). The MaxEnt model provided five feature types: L-linear, Q-quadratic, H-hinge, P-product, and T-threshold. We set the RM range to [0, 4.0], with an increment size of 0.1. Akaike information criterion correction (AICc) was used to assess the fit and complexity of different parameter combinations [39,40]. The area under the receiver operating characteristic curve (AUC) of the test subjects was used to evaluate the ability of different parameter combinations to distinguish between test points and background points. The difference between the training AUC and test AUC values, as well as the 10% training omission rate and minimum training presence omission rate, were evaluated in the fit of different parameter combinations [41]. Finally, the most suitable parameter combination for simulating the data of *C. coreana* was selected. After model optimization, the optimal combination for the suitable habitat model of *C. coreana* was QT feature combination, with a regularization coefficient of 0.8 (Figure 3).

The processed *C. coreana* geographic distribution points data and key environmental variables data were imported into MaxEnt model. The randomly selected train set was “75”, which meant that 75% of the total distribution point data were used as the random sample to train the model, and the other 25% of the total distribution point data were used to test the model predictions. The importance of key environmental variables in model prediction was analyzed using the jack-knife method, with repeated training runs set to “10”, repeated training ruler set to “Subsample”, and the maximum number of iterations set to “5000”.

#### 2.4.2. Evaluation Criteria for MaxEnt Model Accuracy

The evaluation of the prediction results of the MaxEnt model indicated that the test omission rate was closer to the theoretical omission rate, indicating that the accuracy of the constructed model was higher. In this study, the accuracy of the MaxEnt model results was tested by using the AUC value in the receiver operating characteristic curve (ROC) curve analysis method. The value of AUC ranged from 0 to 1. The higher the value of AUC, the better the predictive performance of the model [42]. The accuracy of MaxEnt model predictions was evaluated using the following criterion: the prediction result was unacceptable (fail) when AUC had a value of 0.0–0.6, acceptable (poor) at 0.6–0.7, average (fair) at 0.7–0.8, satisfactory (good) at 0.8–0.9, and very satisfactory at 0.9–1.0 [43].

#### 2.4.3. The Division of the Hierarchy of Suitable Areas

Species distribution points and environmental variables were imported into the MaxEnt model to simulate the habitat suitability index (HSI) for each species. Regions with HSI values greater than the maximum test sensitivity plus specificity logistic threshold were designated as habitats for this species [44]. The suitable habitats for this species were divided into four levels in terms of HSI: 0–0.102 represented unsuitable habitats, 0.102–0.159 represented low-suitable habitats, 0.159–0.412 represented medium-suitable habitats, and 0.412–1 represented highly suitable habitats. Finally, the area sizes of different suitable habitats for *C. coreana* were calculated based on the proportion of different suitable habitats with ArcGIS software 10.8.

## 3. Results

### 3.1. Screening of Distribution Point Data and Identification of Dominant Environmental Factors

**Figure 4:**
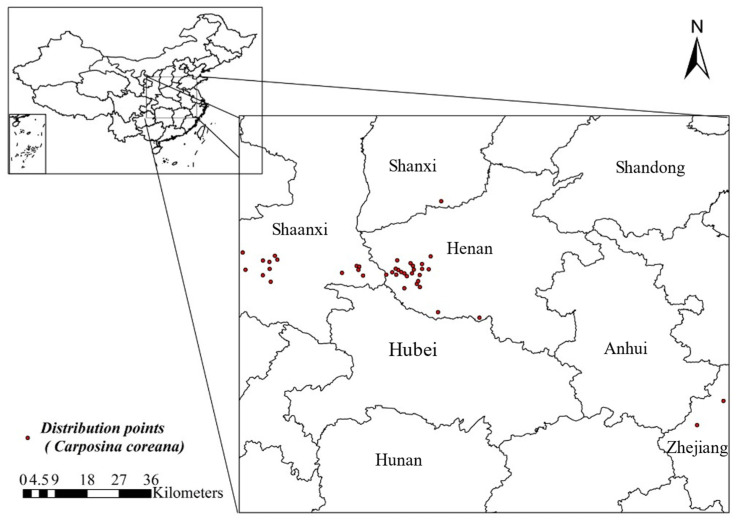
Known geographical distribution records of *Carposina coreana* in China.

Through ArcGIS buffer analysis, 41 distribution points of *C. coreana* were ultimately selected (Figure 4). After correlation analysis, a total of six environmental variables with great influence on *C. coreana* were screened for model prediction to study their current and future distribution (Table 1). The six environmental variables were as follows (Table 1): temperature seasonality (Bio4), minimum temperature in the coldest month (Bio6), mean temperature in coldest quarter (Bio11), precipitation in driest month (Bio14), precipitation in warmest quarter (Bio18), and precipitation in coldest quarter (Bio19).

### 3.2. Verification of Model Accuracy

**Figure 5:**
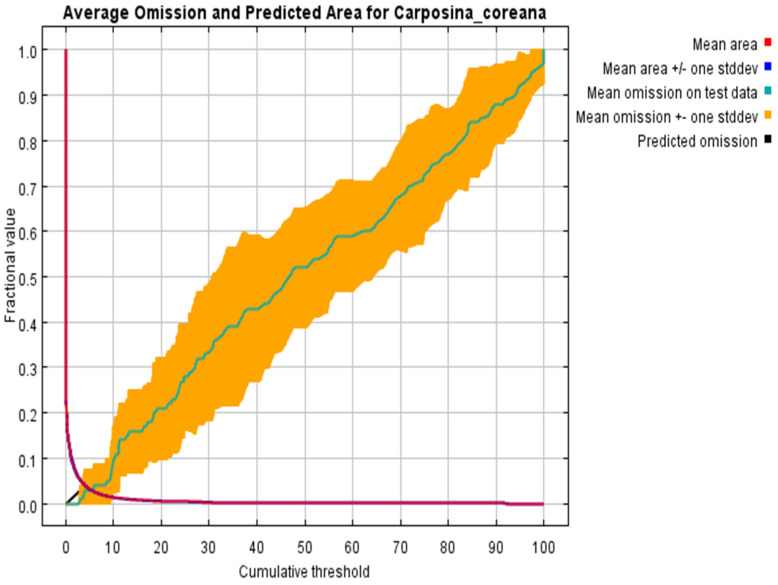
Prediction omission of MaxEnt model.

**Figure 6:**
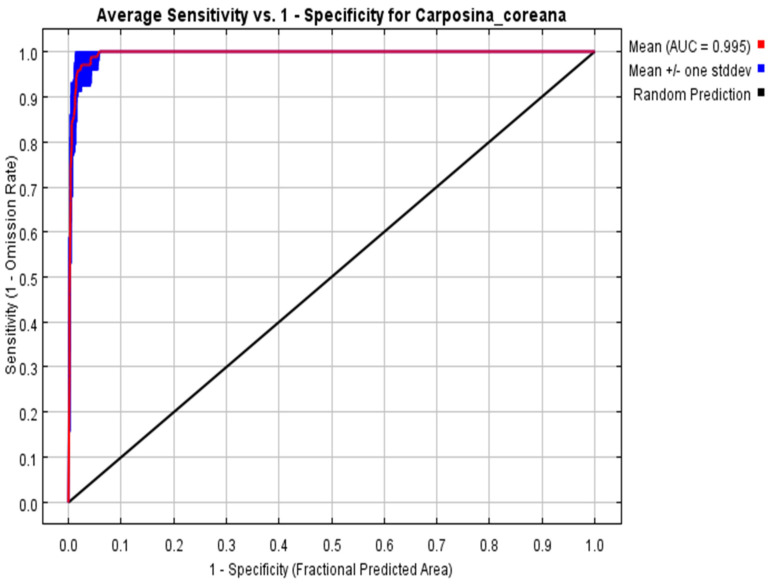
Reliability test of the distribution model created for *Carposina coreana*.

The evaluation of the prediction results of the MaxEnt model showed that the predicted sample omission rate was essentially coincident with the test sample omission rate, indicating that the prediction results of the model were satisfactory (Figure 5). Accuracy tests under the current and future climate scenarios showed that the AUC values were all greater than 0.9, indicating that the prediction results reached the “very good” criterion and could be used to predict suitable habitats for *C. coreana* (Figure 6).

### 3.3. Relationship between the Distribution of Carposina Coreana and Environmental Variables

**Figure 7:**
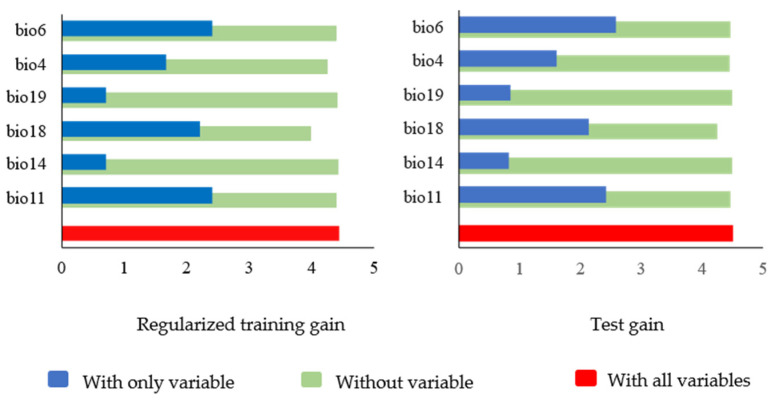
Importance of environmental variables for predicting the distribution of *Carposina coreana*. The blue band represents the importance of the variable to the distribution of the species, and the longer the band, the more important the variable was to the distribution of the species. The green bands represent the degree of specialization of the variable, with shorter bands indicating that the variable contained more unique information and was more likely to affect the distribution of species.

In the model for predicting the suitable habitat of *C. coreana* in China, the selected environmental variables that had a great impact on the distribution of *C. coreana* were analyzed using the jack-knife method. Bio4, Bio6, Bio11, Bio14, Bio18, and Bio19 were the important environmental variables affecting the distribution of *C. coreana* (Figure 7).

Based on the regularization training gain in the MaxEnt model (Figure 7), the highest regularization training gain was observed in relation to the highest annual minimum temperature in the coldest month (Bio6) and mean temperature in the coldest quarter (Bio11), indicating that these variables provided more effective information for the prediction of *C. coreana*. In addition, the shortest was the precipitation in the warmest quarter (Bio18), which indicated that Bio18 had more unique information and was important for species distribution.

**Figure 8:**
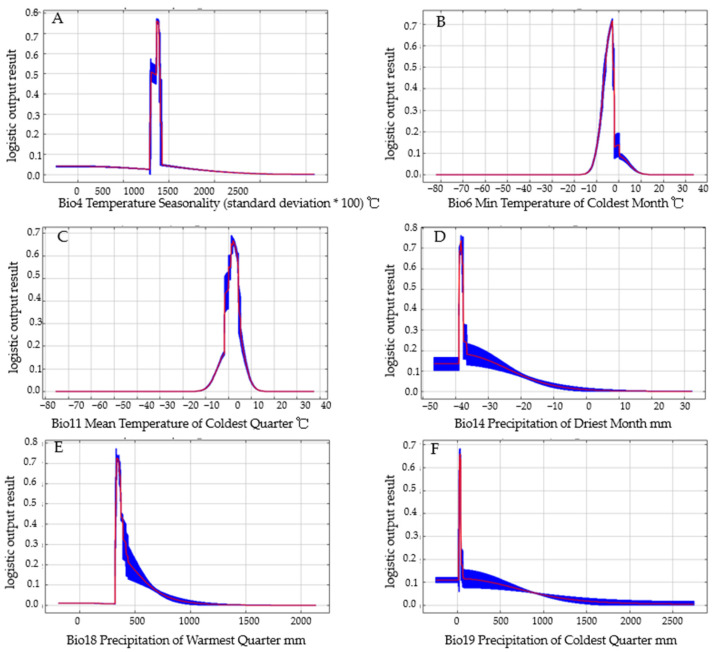
Response curves between probability of presence and environmental variables. (**A**): Bio4 Temperature Seasonality (standard deviation * 100) °C; (**B**) Bio6 Min Temperature of Coldest Month °C; (**C**): Bio11 Mean Temperature of Coldest Quarter °C; (**D**): Bio14 Precipitation of Driest Month mm; (**E**): Bio18 Precipitation of Warmest Quarter mm; (**F**): Bio19 Precipitation of Coldest Quarter mm.

The response curves (Figure 8) created by MaxEnt suggested a high probability of occurrence of *C. coreana* in regions with temperature seasonality (Bio4) of 858.9 to 901.4, a minimum temperature in the coldest month (Bio6) of −6.1 to −2.5 °C, a mean temperature in the coldest quarter (Bio11) of 0.1 to 4.2 °C, precipitation in the driest month (Bio14) of 5.7 to 11.3 mm, precipitation in the warmest quarter (Bio18) of 327.6 to 341.6 mm, precipitation in the coldest quarter (Bio19) of 25.9 to 37.9 mm, and altitude below 700 mm.

Response curves between the probability of presence and environmental variables of *C. coreana* were the same, as shown in the Figure 8. The maximum test sensitivity plus specificity threshold (MTPS) was used as the threshold [44]. There was an optimal value for the effect of temperature seasonality on the probability of existence of *C. coreana*, and this optimal value was 867.4. The temperature seasonality range of suitable species distribution was 858.9–901.4. When the temperature seasonality was 858.9–867.4, the probability of existence increased with an increase in temperature seasonality, and when the temperature seasonality reached 867.4–901.4, the probability of existence decreased with an increase in temperature seasonality (Figure 8A). There was an optimal value for the effect of minimum temperature in the coldest month on the probability of existence of *C. coreana*, and this optimal value was −2.9 °C. The temperature range of suitable species distribution was −6.1–2.5 °C. When the temperature was −6.1–−2.9 °C, the probability of existence increased with an increase in temperature, and when the temperature reached −2.9–2.5 °C, the probability of existence decreased with an increase in temperature (Figure 8B). There was an optimal value for the effect of mean temperature in the coldest quarter on the probability of existence of *C. coreana,* and this optimal value was 1.9 °C. The temperature range of suitable species distribution was 0.1–4.2 °C. When the temperature was 0.1–1.9 °C, the probability of existence increased with an increase in temperature, and when the temperature reached 1.9–4.2 °C, the probability of existence decreased with an increase in temperature (Figure 8C). There was an optimal value for the effect of precipitation in the driest month on the probability of existence of *C. coreana*, and this optimal value was 8.1 mm. The rainfall range of suitable species distribution was 5.7–11.3 mm. When the rainfall was 5.7–8.1 mm, the probability of species increased with an increase in precipitation, and decreased with an increase in precipitation when the rainfall was 8.1–11.3 mm (Figure 8D). There was an optimal value for the effect of precipitation in the warmest quarter on the probability of existence of *C. coreana*, and this optimal value was 341.6 mm. The rainfall range of suitable species distribution was 327.6–371.8 mm. When the rainfall was 327.6–341.6 mm, the probability of species increased with an increase in precipitation, and decreased with an increase in precipitation when the rainfall was 341.6–371.8 mm (Figure 8E). There was an optimal value for the effect of precipitation in the coldest quarter on the probability of existence of *C. coreana*, and this optimal value was 31.9 mm. The rainfall range of suitable species distribution was 25.9–37.9 mm. When the rainfall was 25.9–31.9 mm, the probability of species increased with an increase in precipitation, and decreased with an increase in precipitation when the rainfall was 31.9–37.9 mm (Figure 8F).

### 3.4. Projections under Current Climate Scenarios

**Figure 9:**
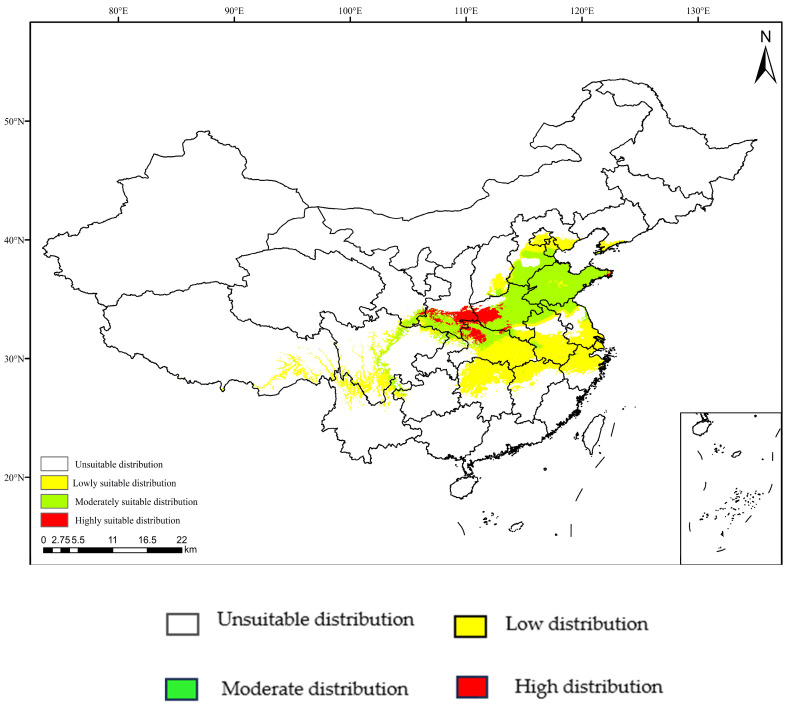
Potential distribution of *Carposina coreana* in China under the current climate conditions.

The MaxEnt model was used to predict the potential suitable range of *C. coreana* in China under the current climate. The results showed that the suitable habitats of *C. coreana* were mainly distributed in most of central China, most of eastern China, part of northern China, and part of northwest China (Figure 9). There was a notable distribution of highly suitable regions in southern Shaanxi, southwestern Henan, and northwestern Hubei. There were more suitable habitat distributions in Shandong, eastern Henan, southern Hebei, and southern Tianjin. Low-suitable habitat distributions were seen in eastern Hubei, northern Hunan, northwestern Jiangxi, southern Anhui, southern Jiangsu, Beijing, northern Tianjin, and northern Zhejiang. Under the current climatic conditions, the total area of suitable habitats for *C. coreana* in China was about 87.71 × 10^4^ km^2^. The area of highly suitable habitats for *C. coreana* was about 7.96 × 10^4^ km^2^, accounting for 9.07% of the total suitable habitats area. The area of moderately suitable habitats for *C. coreana* was about 47.39 × 10^4^ km^2^, accounting for 54.03% of the total suitable habitats area. The area of low-suitable habitats for *C. coreana* was about 32.37 × 10^4^ km^2^, accounting for 36.90% of the total suitable habitats area.

### 3.5. Projections under Future Climate Scenarios

**Figure 10:**
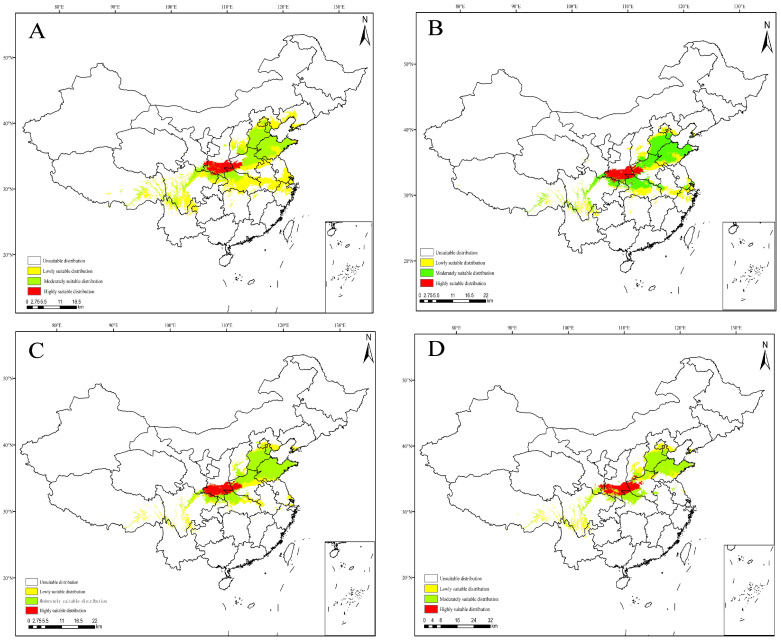
Predicted distribution of *Carposina coreana* in China from 2021 to 2100 under future SSP126 climate scenarios ((**A**) 2021–2040, (**B**) 2041–2060, (**C**) 2061–2080, (**D**) 2081–2100).

**Figure 11:**
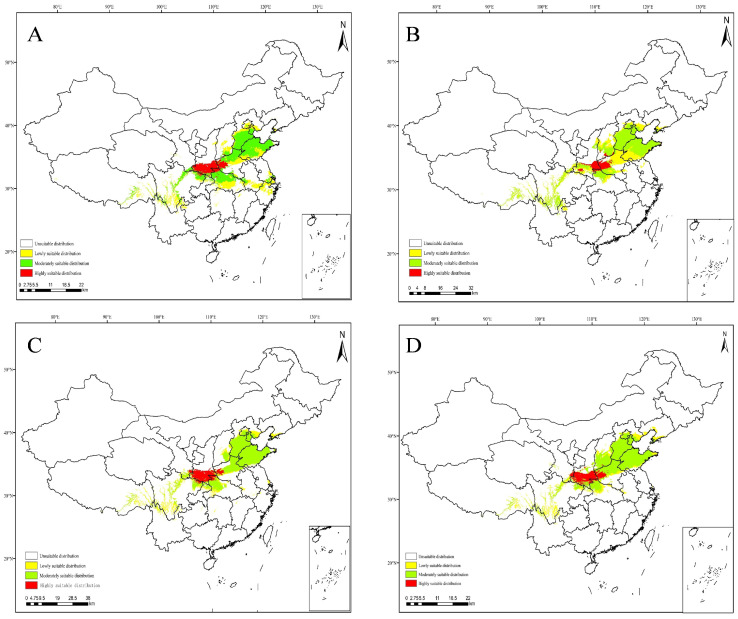
Future species distribution of *Carposina coreana* in China from 2021 to 2100 under SSP245 predicted climate scenarios ((**A**) 2021–2040, (**B**) 2041–2060, (**C**) 2061–2080, (**D**) 2081–2100).

**Figure 12:**
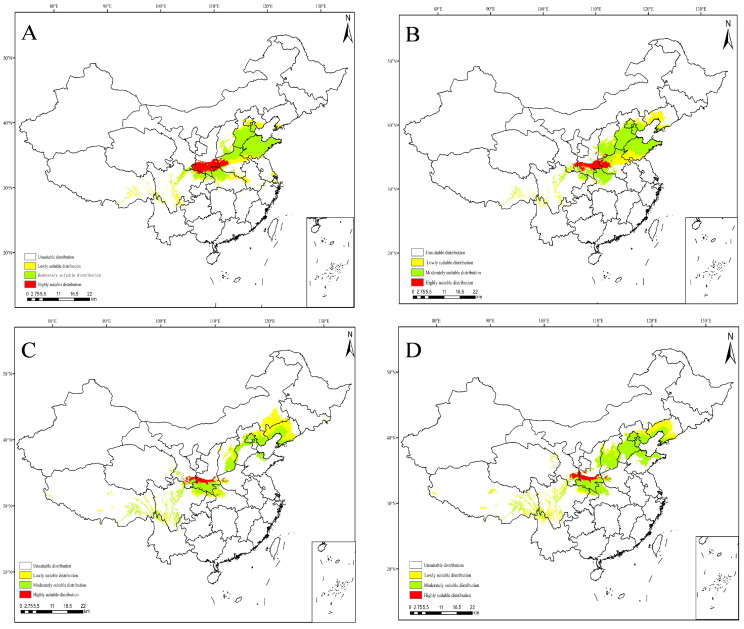
Future species distribution of *Carposina coreana* in China from 2021 to 2100 under SSP370 predicted climate scenarios ((**A**) 2021–2040, (**B**) 2041–2060, (**C**) 2061–2080, (**D**) 2081–2100).

**Figure 13:**
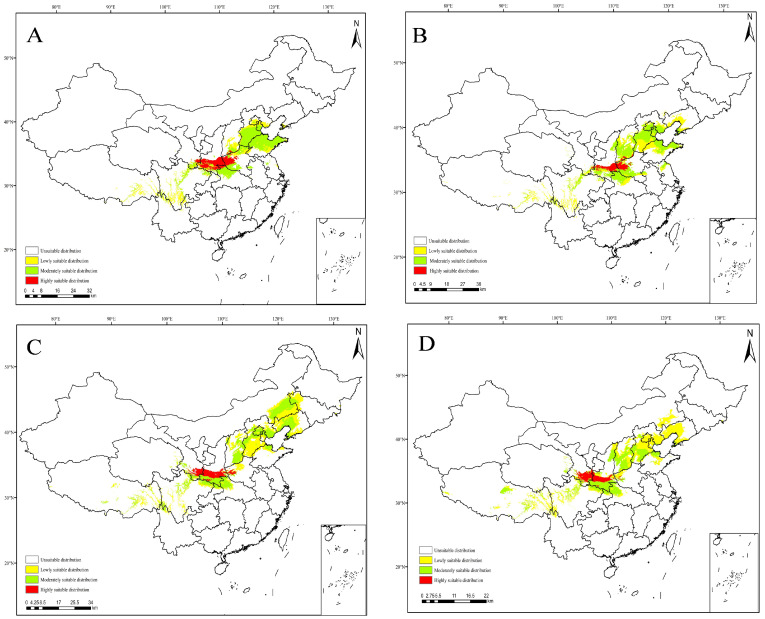
Future species distribution of *Carposina coreana* in China from 2021 to 2100 under SSP585 predicted climate scenarios ((**A**) 2021–2040, (**B**) 2041–2060, (**C**) 2061–2080, (**D**) 2081–2100).

The MaxEnt model was used to predict the potential suitable range of *C. coreana* in China under the future climate scenario. The results showed that the boundary of the suitable habitats of *C. coreana* tended to shift to northward compared with that under the current climate scenario (Figure 10, Figure 11, Figure 12 and Figure 13).

**Figure 14:**
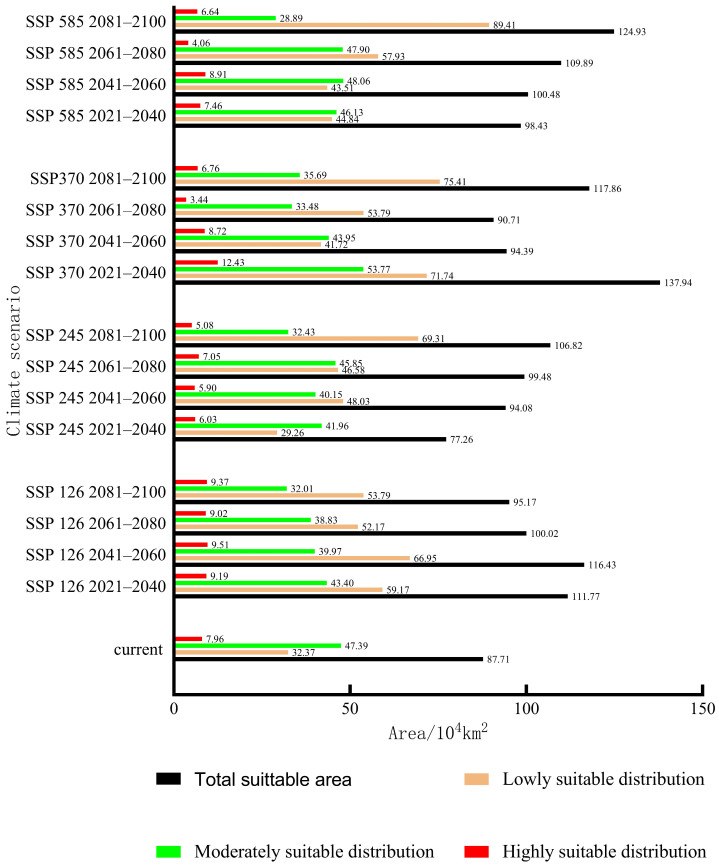
Area prediction of *Carposina coreana* in China under current and future climate scenarios.

The total area of the suitable habitats of *C. coreana* under the current climate scenario was about 87.71 × 10^4^ km^2^, while the total suitable area increased to 137.94 × 10^4^ km^2^ under the 2021–2040 SSP370 climate scenario, and the total area of the suitable area decreased to 77.26 × 10^4^ km^2^ under the 2021–2040 SSP245 climate scenario. Under the current climate scenario, the area of low-level suitable habitats was 32.37 × 10^4^ km^2^; the area of low-level suitable habitats under the SSP245 2021–2040 climate scenario decreased to 29.26 × 10^4^ km^2^, and the area of low-level suitable habitats increased to 89.41 × 10^4^ km^2^ under the SSP585 2081–2100 climate scenario. The area of moderately suitable habitats was 47.39 × 10^4^ km^2^ under the pre-conditional climate scenario, which decreased to 28.89 × 10^4^ km^2^ under the 2081–2100 SSP585 climate scenario, and increased to 53.77 × 10^4^ km^2^ under the 2021–2040 SSP370 climate scenario. Under the current climate scenario, the area of highly suitable habitats was 7.96 × 10^4^ km^2^; the area of highly suitable habitats under the 2041–2060 SSP370 climate scenario decreased to 3.44 × 10^4^ km^2^, and the area of highly suitable habitats increased to 12.43 × 10^4^ km^2^ under the 2021–2040 SSP370 climate scenario (Figure 14).

## 4. Discussion

In this study, the MaxEnt model was used to predict current and future suitable habitat areas for *C. coreana*. As an algorithmic model, MaxEnt tends to build very complex functional relationships to fit observational data [45]. This is also an important reason supporting why the maximum entropy model predicts better. The three main factors that affect the complexity of MaxEnt models include the number of environmental variables modeled, the functional pattern, and the regularization multiplier [46]. The accuracy of the model prediction is measured by the size of the AUC value, and the AUC value in this study was 0.995, which showed that the model had good accuracy and could be used for prediction of suitable regions.

The key environmental variables affecting the distribution of *C. coreana* were determined by the knife-cut method combined with the biological characteristics of *C. coreana*. The effects of precipitation in the warmest quarter (Bio18), temperature seasonality (Bio4), the minimum temperature in the coldest month (Bio6), and the mean temperature in the coldest quarter (Bio11) reached 90.4%. A possible reason for this is that *C. coreana* produces one generation a year and overwinters as mature larvae. From the end of July to early August of the second year, the mature larvae emerge as adults [4,5]. On the one hand, *C. coreana* lays eggs and mates during the warmest season, and the precipitation during the warmest season affects the population and eclosion of *C. coreana*. On the other hand, the lowest temperature in the coldest month affects the survival rate of *C. coreana* overwintering cocoons.

In the present study, the MaxEnt model was used for the first time to analyze the distribution of suitable habitats of *C. coreana* in China. The results showed that suitable habitat areas for *C. coreana* under the current climate scenarios were mainly distributed in Shaanxi, Henan, and Hubei, followed by the Shanxi province, with weaker distribution in the Zhejiang and Anhui provinces, which were different from those found by Bai et al. [47]. The inverse distance weight (IDW) method was used to construct a distribution model of *C. coreana* by Bai [47], predicting that the highly suitable areas only included Henan and Hubei, while the weaker distributed regions were Shaanxi and Shanxi. The possible reasons for the differences might be the different prediction models and sampling points.

Research has shown that species distributions are linked to climate change, with northern shifts in a species’ suitable zone consistent with rising local winter temperatures [48]. The results of this study on the prediction of the suitable habitat areas for *C. coreana* showed that the total area of suitable habitats for *C. coreana* would increase under the influence of global warming, which was consistent with other studies. In the prediction of suitable habitat areas for *Brachyponera nigrita* Emery, 1895 (Hymenoptera: Formicidae) based on future climate scenarios (RCP4.5 and RCP8.5), the moderately suitable and highly suitable areas both increased [49]. Under the SSP24.5 scenario (2050s), the area of suitable habitat for *Batocera horsfieldi* (Hope), 1839 (Coleoptera: Cerambycidae) was found to increase significantly [50].

Compared with the total suitable area for *C. coreana* under the current climate scenario, the total suitable area increased by 57.23% under the 2021–2040 SSP370 climate scenario, and the total suitable area decreased by 11.91% under the 2021–2040 SSP245 climate scenario. Compared with the area of highly suitable habitats for *C. coreana* under the current climate scenario, the area of highly suitable habitats under the 2021–2040 SSP370 climate scenario increased by 56.15%, and the area of highly suitable habitats under the 2061–2080 SSP370 climate scenario decreased by 56.65%. Compared with the area of moderately suitable habitats for *C. coreana* under the current climate scenario, the area of moderately suitable habitats under the 2021–2040 SSP370 climate scenario increased by 13.46%, and the area of moderate suitable habitats under the 2081–2100 SSP585 climate scenario decreased by 39.01%. Compared with the area of low-level suitable habitats of *C. coreana* under the current climate scenario, the area of low-level suitable habitats under the 2081–2100 SSP585 climate scenario increased by 176.21%, and the area of the low-level suitable habitats under the 2021–2040 SSP245 climate scenario decreased by 9.57%. Under the SSP370 climate scenario, the area of moderately suitable habitats and highly suitable habitats increased rapidly, which was not suitable for the prediction of suitable habitats for *C. coreana*. The rapid growth of the area of low-grade suitable habitats under the 2081–2100 SSP585 climate scenario was not consistent with the distribution trend of *C. coreana*. Under the 2061–2080 SSP370 climate scenario, the area of highly suitable habitats decreased too rapidly, and it was not suitable for prediction of suitable habitat areas for *C. coreana*. Under the SSP126 climate scenario, there was no rapid growth in the low, medium, and high-suitable areas, which was consistent with the distribution of *C. coreana*. Bai et al. [43] used GIS technology to predict the distribution of *C. coreana*, and their study showed that *C. coreana* would be distributed in Henan, Hubei, Shaanxi, Anhui, Zhejiang, and Shanxi, and there was a trend of transmission between different production areas. Zhang conducted a study on the suitable growth area of *C. coreana* through MaxEnt and ArcGIS software, and found that the most suitable areas for *C. coreana* growth were distributed in Henan, Shaanxi, Zhejiang, Chongqing, Hubei, Sichuan, Anhui, Hunan, Shandong, and other places [51], which overlapped with the suitable growth area of *C. coreana* in this study. Attention should be paid to the spread of *C. officinalis* during dogwood transplanting and *C. officinalis* fresh fruit transportation.

At the same time, only the corresponding environmental variables were selected, and the MaxEnt model was used to predict suitable habitats for *C. coreana* in China. The distribution of species is not only be limited by environmental conditions, but also affected by other factors, such as interspecific relationships, management measures, natural enemies, and socio-economic development levels [52,53]. Therefore, in the future, when studying suitable habitat areas for *C. coreana*, biological and abiotic factors, such as human activities and host type, can be incorporated into the model, and more accurate prediction results will be obtained.

## 5. Conclusions

This paper is the first study to predict the distribution of *C. coreana* under current and future climate scenarios. The AUC value predicted by the model indicated the accuracy of the prediction results. The results showed that precipitation in the warmest quarter (Bio18), temperature seasonality (Bio4), the minimum temperature in the coldest month (Bio6), and the mean temperature in the coldest quarter (Bio11) were the dominant environmental variables affecting the distribution of *C. coreana*. The suitable areas for *C. coreana* under the current climate scenarios were mainly distributed in central China, and the highly suitable areas were distributed in southern Shaanxi, southwestern Henan, and northwestern Hubei. Under future climate scenarios, the boundaries of the suitable areas for *C. coreana* tended to shift to northern China. Future increases in temperature would provide favorable conditions for the expansion of the suitable growth area for *C. coreana*.

## Figures and Tables

**Table 1:** Environmental variables affecting *Carposina coreana* distribution and their contribution rates.

No.	Climatic Variable	Contribution Rate %
Bio4	Temperature Seasonality (standard deviation * 100)	26.3
Bio6	Minimum Temperature in Coldest Month	14.5
Bio11	Mean Temperature in Coldest Quarter	14.1
Bio14	Precipitation in Driest Month	0.5
Bio18	Precipitation in Warmest Quarter	35.5
Bio19	Precipitation in Coldest Quarter	9.1

## Data Availability

Data are available upon request from the authors.
